# The bidirectional regulatory network between ATF4 and lncRNAs in systemic diseases

**DOI:** 10.3389/fonc.2025.1562861

**Published:** 2025-07-24

**Authors:** Dongdong Wu, Mei Huang, Changning Ma, Xuetong Xu, Tianhui Wu, Miao Zhang

**Affiliations:** ^1^ College of Physical Education, Yanshan University, Qinhuangdao, China; ^2^ School of Rehabilitation Medicine, Wenzhou Medical University, Wenzhou, China; ^3^ State Key Laboratory of Metastable Materials Science and Technology, Hebei Key Laboratory of Applied Chemistry, Yanshan University, Qinhuangdao, China

**Keywords:** lncRNA, ATF4, systemic diseases, molecular targets, human health

## Abstract

Long non-coding RNAs (lncRNAs) are pivotal regulators of gene expression across multiple biological contexts, including stress responses and cellular adaptation. Activating transcription factor 4 (ATF4) is a key transcriptional effector of the integrated stress response (ISR), modulating genes involved in redox balance, amino acid metabolism, autophagy, and apoptosis. Emerging evidence has uncovered complex interactions between ATF4 and lncRNAs in systemic diseases, where lncRNAs can act as either downstream targets or upstream modulators of ATF4 signaling. This bidirectional crosstalk influences critical processes such as tumor progression, metabolic reprogramming, immune evasion, and skeletal homeostasis. In this review, we comprehensively summarize the regulatory roles of ATF4–lncRNA interactions in four major physiological systems: digestive, respiratory, immune, and skeletal. Furthermore, we highlight the therapeutic potential of selectively targeting these lncRNAs to modulate ATF4-mediated stress responses in a disease- and context-dependent manner. Our insights provide a conceptual framework and translational perspective for future research and precision therapies targeting the ATF4–lncRNA regulatory axis.

## Introduction

LncRNAs are transcripts longer than 200 nucleotides that lack protein-coding potential but exert essential regulatory roles in various biological processes. Transcribed by RNA polymerase II and often spliced, capped, and polyadenylated, lncRNAs function at multiple levels of gene regulation—including chromatin remodeling, transcriptional activation or repression, mRNA stability, and translation control (1–3). They are involved in fundamental physiological activities such as development, metabolism, and cell differentiation, and have been increasingly recognized as critical regulators in the pathogenesis of systemic diseases, including cancer, immune dysfunction, fibrosis, and metabolic syndromes (1, 2).

**Figure 1:**
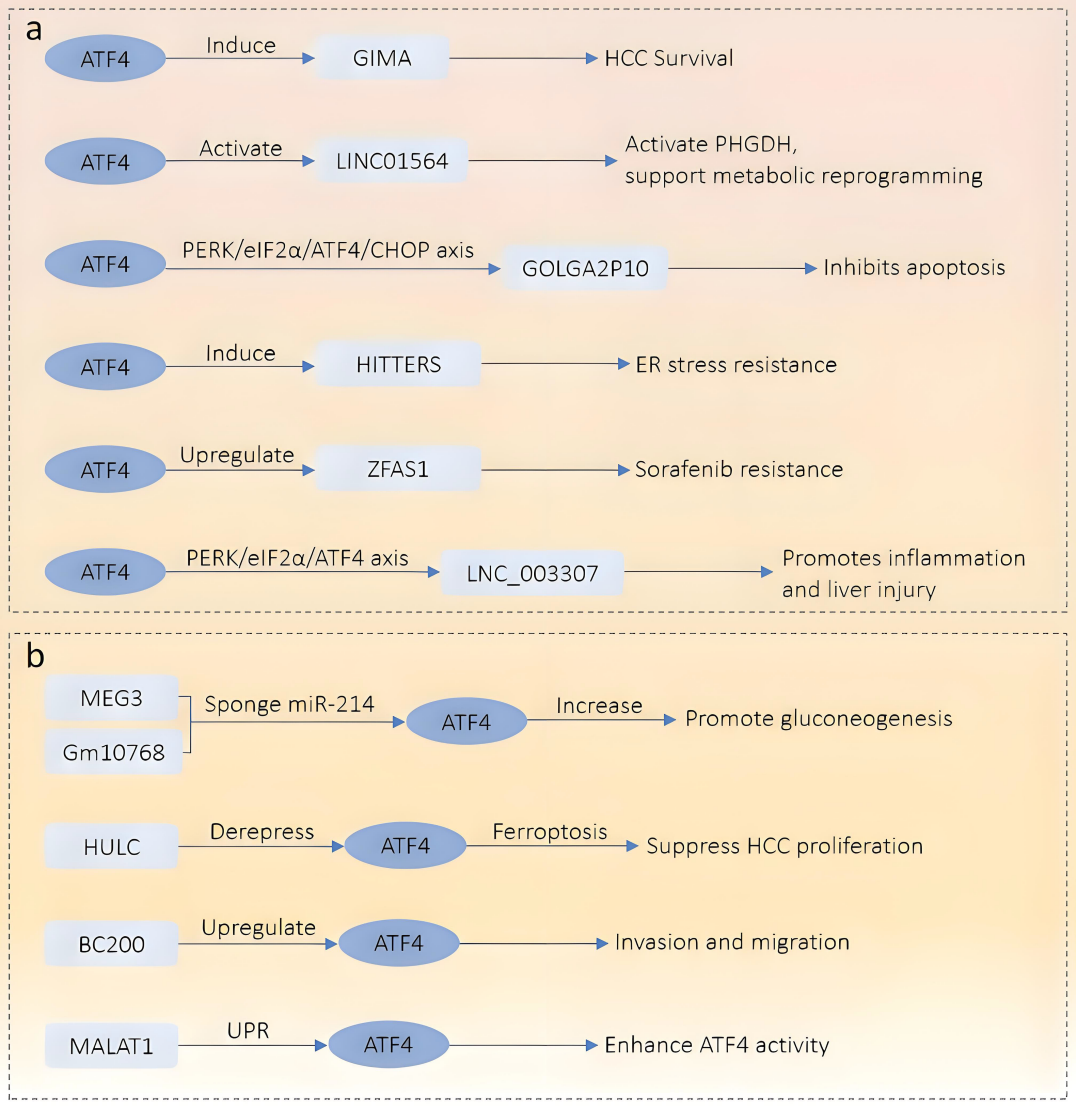
Schematic overview of the regulatory network between ATF4 and lncRNAs in the digestive system. **(a)** Downstream lncRNAs regulated by ATF4. ATF4 activates multiple stress-responsive lncRNAs under various pathological conditions. These include GIMA (promotes HCC survival), LINC01564 (activates PHGDH and metabolic reprogramming), GOLGA2P10 (via PERK/eIF2α/ATF4/CHOP axis, inhibits apoptosis), HITTERS (enhances ER stress resistance), ZFAS1 (confers sorafenib resistance), and LNC_003307 (inflammatory liver injury via PERK/eIF2α/ATF4 axis). **(b)** Upstream lncRNAs that regulate ATF4 expression. MEG3 and Gm10768 act as competing endogenous RNAs (ceRNAs) to sponge miR-214, thereby increasing ATF4 expression and promoting gluconeogenesis. HULC derepresses ATF4, promoting ferroptosis and inhibiting HCC proliferation. BC200 upregulates ATF4 to enhance invasion and migration. MALAT1 enhances ATF4 activity through the unfolded protein response (UPR) pathway.

**Figure 2:**
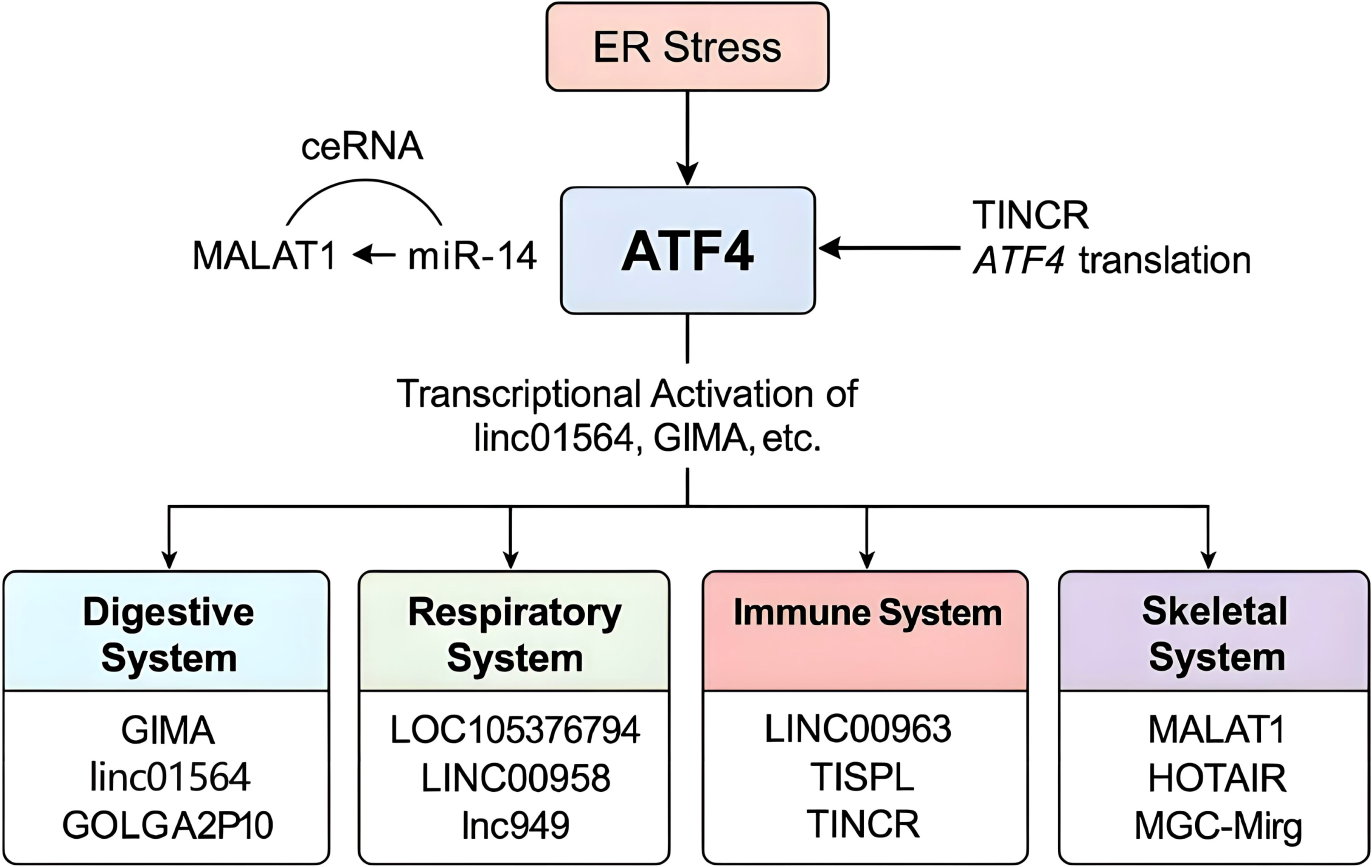
Mechanistic overview of the ATF4–lncRNA regulatory network. ATF4 is activated by ER stress and transcriptionally regulates various lncRNAs in a system-specific manner. Simultaneously, multiple lncRNAs can influence ATF4 expression or translation through ceRNA activity (e.g., MALAT1–miR-214–ATF4 axis) or direct inhibition (e.g., TINCR). Systems are color-coded, and representative lncRNAs involved in each are highlighted.

ATF4 is a basic leucine zipper (bZIP) transcription factor and a key downstream effector of the ISR. Under non-stressed conditions, translation of ATF4 is repressed by upstream open reading frames (uORFs) in its 5′ untranslated region. In response to cellular stress—such as amino acid deprivation, oxidative damage, or endoplasmic reticulum (ER) stress—the PERK–eIF2α–ATF4 signaling axis is activated (4–6), enabling selective ATF4 translation. Once expressed, ATF4 controls a wide range of target genes involved in redox homeostasis, amino acid metabolism, autophagy, and apoptosis, thereby helping cells adapt to or eliminate stress-induced damage (5, 7). The ceRNA (competing endogenous RNA) hypothesis, first proposed in 2011, describes how RNA transcripts—including lncRNAs, mRNAs, and circular RNAs (circRNAs)—compete for shared miRNA response elements (MREs), thereby modulating each other’s expression (8). This regulatory model has been validated in various pathophysiological contexts, including cancer and metabolic disorders (9). Recent studies have uncovered a complex bidirectional regulatory network between ATF4 and lncRNAs. In one direction, ATF4 transcriptionally induces several stress-responsive lncRNAs such as GOLGA2P10 and linc01564, which contribute to hepatocellular carcinoma (HCC) survival and metabolic reprogramming (10, 11). In the opposite direction, lncRNAs such as MEG3 and HULC modulate ATF4 expression through post-transcriptional mechanisms, particularly via microRNA (miRNA) sponging within the ceRNA framework (12, 13). For instance, lncRNAs such as Gm10768 and LOC105376794 regulate ATF4 by sequestering miRNAs that would otherwise inhibit its translation (14, 15). In this review, we systematically explore the regulatory mechanisms connecting ATF4 and lncRNAs across four physiological systems: digestive, respiratory, immune, and skeletal. To ensure conceptual clarity, we categorize the reported interactions into two groups: (1) lncRNAs regulated by ATF4, and (2) lncRNAs that regulate ATF4. These findings are summarized in **Tables 1A** and **1B** and visually integrated in **Figures 1** and **2**. This framework provides a clearer mechanistic basis for understanding how the ATF4–lncRNA axis contributes to disease pathogenesis.

**Table 1A:** lncRNAs regulated by ATF4 (ATF4 → lncRNA).

lncRNA	Disease/Model	Regulation mechanism	Effect/Outcome	Reference
GIMA	HCC	Induced under glucose deprivation	Enhances autophagy, promotes survival	(16)
LINC01564	HCC	Transcriptionally activated by ATF4	Activates PHGDH, supports metabolic reprogramming	(11)
GOLGA2P10	HCC	PERK/eIF2α/ATF4/CHOP axis	Inhibits apoptosis via Bcl-2 regulation	(10)
HITTERS	OSCC	ATF4-inducible under ER stress	Promotes DNA repair, ER stress resistance	(17)
ZFAS1	HCC	ATF4-dependent expression	Confers sorafenib resistance	(18)
LNC_003307	Inflammatory liver injury (LPS model)	PERK/eIF2α/ATF4 axis	Promotes inflammation and liver injury	(19)
LINC00958	LUAD	Activated by MYC and ATF4	Promotes oncogenic transcription and proliferation	(22)
lnc949	Pulmonary fibrosis (bleomycin model)	ATF4 target	Attenuates EMT and fibrosis via TGF-β/JNK signaling	(23)
AC079466.1	NSCLC (ER stress model)	Induced by ATF4	Promotes apoptosis	(24)
MIR155HG	Macrophages (LPS model)	PERK–eIF2α–ATF4 axis	Increases miR-155 and inflammatory cytokine expression	(30)
TISPL	General stress response	Direct ATF4 target	Associated with IL-6, HMOX1 induction	(31)
MGC-Mirg	KOA model	Induced by PERK–ATF4 signaling	Mediates chondrocyte degeneration under ER stress	(35, 36)
H19	Osteogenesis	Upregulated via Wnt–ATF4	Promotes osteogenic differentiation	(37)
lnc-OAD	Adipogenesis	ATF4-induced via Wnt pathway	Modulates mitotic clonal expansion	(38)

**Table 1B:** lncRNAs that regulate ATF4 (lncRNA → ATF4).

lncRNA	Disease/Model	Regulation mechanism	Effect/Outcome	Reference
MEG3	Diabetes mellitus	Sponges miR-214	Increases ATF4, promotes gluconeogenesis	(12)
Gm10768	Hepatic gluconeogenesis	Sponges miR-214	Enhances ATF4, increases glucose output	(14)
HULC	HCC	Sponges miR-3200-5p	Induces ATF4-mediated ferroptosis	(13)
BC200	ESCC	Upregulates ATF4	Enhances invasion and migration	(20)
MALAT1	CRC	ER stress-induced via PERK/IRE1–ATF4	Enhances ATF4 signaling	(21)
LINC01278	NSCLC	Sponges miR-877-5p	Restores ATF4 expression, promotes tumor growth	(25)
LOC105376794	EGFR-mutant LUAD	Promotes ATF4/CHOP signaling	Enhances proliferation and TKI resistance	(15)
OIP5-AS1	Sepsis-induced ALI	Stabilizes ATF4 mRNA	Promotes inflammatory injury	(26)
lnc-HFE2-2:1	NSCLC (celecoxib treated)	Positively correlated with ATF4	Activates ER stress, promotes apoptosis	(27)
TUG1	Diabetes-related inflammation	Positively correlated with ATF4	Suggests involvement in ER stress regulation	(32)
Lnc-DC	Dendritic cell maturation	Indirect via STAT3	Contributes to immune activation	(33)
TINCR	Melanoma	Binds ATF4 5′UTR, inhibits translation	Prevents immune evasion and stress resistance	(34)
DANCR	Osteogenesis	Directly suppresses ATF4	Inhibits osteoblast differentiation	(39)
HOTAIR	Osteogenesis	Sponges miR-214	Upregulates ATF4, promotes osteoblast function	(40)
MALAT1	SANFH	Sponges miR-214	Increases ATF4, supports osteogenesis	(41)
KCNQ1OT1	Osteoblast differentiation	via miR-205-5p/RICTOR	Indirectly reduces ATF4, impairs bone formation	(42)

To visually summarize the key mechanisms underlying ATF4–lncRNA interactions across different systems, we present an integrative schematic (**Figure 2**). This diagram highlights representative lncRNAs that are either regulated by ATF4 or modulate ATF4 expression, illustrating their roles in disease progression via ER stress, oxidative signaling, or miRNA-mediated ceRNA activity.

## ATF4–lncRNA interactions in digestive system diseases

The digestive system—particularly the liver, pancreas, and gastrointestinal tract—is highly susceptible to various stress stimuli, including metabolic overload, inflammation, and ER stress. These perturbations often activate the ISR, with ATF4 acting as a central transcriptional effector. Growing evidence indicates that ATF4 interacts with diverse lncRNAs in the digestive system, forming a bidirectional regulatory network that affects tumor growth, inflammation, gluconeogenesis, and treatment resistance.

ATF4 transcriptionally activates several stress-responsive lncRNAs in HCC and other liver-related diseases. Under glucose-deprivation stress, ATF4 directly induces GIMA, which enhances autophagy and promotes HCC cell survival by maintaining intracellular redox balance (16). Similarly, LINC01564 is upregulated by ATF4 and activates phosphoglycerate dehydrogenase (PHGDH), facilitating serine biosynthesis and metabolic reprogramming in liver cancer cells (11). These metabolic adaptations support tumor cell proliferation under nutrient stress. In parallel, GOLGA2P10 is transcriptionally activated through the PERK–eIF2α–ATF4–CHOP axis and inhibits apoptosis by regulating Bcl-2 family proteins (10).

In oral squamous cell carcinoma (OSCC), another ATF4-inducible lncRNA, HITTERS, was shown to enhance DNA repair capacity by stabilizing the MRE11–RAD50–NBS1 complex, enabling cells to resist ER stress-induced damage (17). In drug resistance contexts, ZFAS1 has been reported to be upregulated by ATF4 and contributes to sorafenib resistance in HCC by suppressing apoptotic signaling (18). Furthermore, LNC_003307 exacerbates inflammatory liver injury in lipopolysaccharide (LPS)-challenged models through the PERK/eIF2α/ATF4 signaling cascade (19).

Conversely, several lncRNAs act upstream to regulate ATF4 expression via post-transcriptional mechanisms, especially through microRNA sponging. The lncRNA MEG3 has been shown to function as a ceRNA by sponging miR-214, a negative regulator of ATF4. This leads to increased ATF4 levels, which in turn elevate the expression of gluconeogenic transcription factors such as FoxO1, PEPCK, and G6Pc, ultimately promoting hepatic glucose production in insulin-resistant states (12). Likewise, Gm10768 enhances hepatic gluconeogenesis via the same miR-214–ATF4 axis, further reinforcing the metabolic impact of lncRNA–ATF4 interaction in glucose homeostasis (14).

In the context of ferroptosis, the lncRNA HULC sequesters miR-3200-5p, thereby de-repressing ATF4 and activating ferroptotic pathways that suppress HCC proliferation (13). In esophageal squamous cell carcinoma (ESCC), BC200 was shown to upregulate ATF4 expression, enhancing the migration and invasion of cancer cells under nutrient-stressed conditions (20). Additionally, MALAT1, a well-characterized lncRNA upregulated during ER stress, is known to enhance ATF4 activity through the PERK/eIF2α and IRE1/XBP1 branches of the unfolded protein response (UPR), particularly in colorectal cancer (CRC) models (21).

These findings collectively underscore the complexity of ATF4–lncRNA interactions in digestive system diseases. ATF4 functions as a transcriptional activator of several lncRNAs in response to cellular stress, thereby modulating processes such as autophagy, metabolic adaptation, ER stress resistance, and drug sensitivity. Conversely, multiple lncRNAs regulate ATF4 expression or translation through post-transcriptional mechanisms, most commonly via microRNA sponging.

In the digestive system, the ATF4–lncRNA axis orchestrates a dynamic stress-adaptive network that regulates key pathological processes such as tumor growth, metabolic reprogramming, ER stress tolerance, and drug resistance. ATF4 transcriptionally activates several lncRNAs in hepatocellular and gastrointestinal cancers, while numerous lncRNAs modulate ATF4 expression via miRNA-mediated mechanisms. This bidirectional regulation reflects a tightly controlled feedback system, positioning ATF4–lncRNA interactions as potential targets for therapeutic intervention in digestive diseases.

## ATF4–lncRNA interactions in respiratory system diseases

The lungs are continuously exposed to various environmental insults such as air pollutants, pathogens, allergens, and hypoxia, which can lead to oxidative stress, ER stress, and inflammation. These stressors activate the ISR, where ATF4 acts as a master regulator that determines cell fate by inducing genes involved in adaptation, autophagy, or apoptosis. LncRNAs have recently emerged as pivotal regulators of ATF4 signaling in respiratory diseases, including lung adenocarcinoma (LUAD), non-small cell lung cancer (NSCLC), pulmonary fibrosis and acute lung injury (ALI).

Several lncRNAs are transcriptionally upregulated by ATF4 in response to stress. LINC00958, activated by MYC and ATF4 in LUAD, promotes oncogenic transcription programs involving HOXA1, NANOG, and FOSL2, and contributes to cell proliferation and poor prognosis (22). In bleomycin-induced pulmonary fibrosis, the ATF4-responsive lnc949 alleviates fibrotic remodeling by modulating TGF-β/Smad2/3 and JNK pathways, thus reducing Epithelial–Mesenchymal Transition (EMT) and collagen deposition (23). Similarly, AC079466.1 is induced during ER stress and mediates ATF4-dependent apoptosis in NSCLC cells, offering potential therapeutic value (24). Also in NSCLC, LINC01278 could directly bind to miR-877-5p. Then miR-877-5p targeted ATF4. ATF4 upregulation could partly restore the carcinogenic effect of LINC01278 *in vitro* and *in vivo* (25).

Conversely, several lncRNAs act upstream of ATF4 to regulate its expression or stability. LOC105376794 is significantly elevated in EGFR-mutant LUAD and promotes ATF4/CHOP signaling, contributing to proliferation, migration, and resistance to tyrosine kinase inhibitors (TKIs) (15). Emerging evidence suggests that lncRNA OIP5-AS1 modulates ATF4 signaling in sepsis-induced ALI. In LPS-stimulated 16HBE cells, miR-338-3p suppresses OIP5-AS1 expression, which normally stabilizes ATF4 mRNA, thereby reducing ATF4-mediated cell injury. This regulatory axis highlights the role of lncRNA–ATF4 networks in the inflammatory and apoptotic processes of sepsis-related ALI (26).

Furthermore, in NSCLC, Celecoxib promotes ATF4 expression by upregulating Inc-HFE2-2:1, thereby activating the ER stress to enhance tumor cell apoptosis (27). Finally, an integrative transcriptomic analysis of NSCLC patient tissues identified a panel of lncRNAs including LINC01547, which were co-expressed with ATF4, suggesting broader lncRNA–ATF4 co-regulatory networks (28).

In respiratory diseases, the ATF4–lncRNA axis plays a central role in coordinating cellular stress responses, inflammation, and tumor progression. Stress-inducible lncRNAs such as LINC00958, lnc949, and AC079466.1 act as downstream effectors of ATF4, modulating fibrosis, apoptosis, and oncogenic signaling. Conversely, upstream regulators like OIP5-AS1, LOC105376794, and LINC01278 fine-tune ATF4 expression through miRNA interactions and mRNA stabilization. These bidirectional interactions suggest that lncRNAs serve as both mediators and modulators of ATF4-driven pathophysiological processes in the lung, offering promising targets for therapeutic intervention in lung cancer, fibrosis, and inflammatory injury.

## ATF4–lncRNA interactions in immune system diseases

The immune system plays a central role in maintaining organismal homeostasis and defending against pathogens, but its dysregulation is also closely associated with chronic inflammation, autoimmune diseases, and tumor immune evasion. ATF4, a key transcriptional regulator of the ISR, is increasingly recognized as a mediator of immune homeostasis, especially under conditions of oxidative stress, nutrient deprivation, and cytokine stimulation. Recent studies have demonstrated that lncRNAs are crucial components in modulating ATF4 activity and orchestrating immune signaling networks.

In pancreatic cancer, evidence suggests that the traditional Chinese medicine Qingyihuaji formula (QYHJ) can inhibit pancreatic cancer progression by modulating the LINC00346–OMA1–ATF4 signaling axis. Specifically, QYHJ appears to suppress LINC00346 expression and activate the OMA1–ATF4 pathway, which may represent a promising therapeutic strategy for pancreatic cancer treatment (29). Additionally, MIR155HG is upregulated by the PERK–eIF2α–ATF4 axis in macrophages exposed to LPS, leading to increased miR-155 expression and amplification of proinflammatory cytokine responses (30). A study also found that the expression of the novel RNA transcript TISPL is regulated by ATF4 and is significantly upregulated under various stress conditions that activate ATF4. Its expression pattern is closely related to the activation of ATF4. TISPL may serve as a biomarker for detecting stress conditions that activate ATF4, providing a new perspective and tool for studying cellular stress responses (31).

Several lncRNAs act upstream of ATF4 to fine-tune its expression or function during immune regulation. For example, in diabetes, the expression of TUG1 was significantly increased. TUG1 expression showed a positive correlation with ATF4 expression, as well as with indices of glycemic control and markers of ER stress. This suggests that TUG1 may be associated with ATF4 in the context of ER stress related to hyperglycemia (32). In dendritic cells, Lnc-DC interacts with ATF4 indirectly through STAT3, contributing to cytokine release and T cell activation (33).

In addition to these classical immune modulatory roles, lncRNAs may also influence tumor–immune dynamics through ATF4 regulation. A recent study identified TINCR as a suppressor of metastatic melanoma dissemination via direct inhibition of ATF4 translation. Mechanistically, TINCR binds to the 5′ untranslated region (UTR) of ATF4 mRNA, blocking ribosome loading and reducing ATF4 protein expression. Loss of TINCR leads to stress-induced ATF4 activation, immune evasion, and resistance to BRAF/MEK inhibitors in melanoma cells, highlighting its role at the intersection of immune regulation and tumor progression (34).

Emerging evidence underscores the intricate interplay between ATF4 and lncRNAs in shaping immune responses. ATF4 not only transcriptionally regulates immune-related lncRNAs such as MIR155HG and TISPL under stress conditions but is also modulated by lncRNAs like TUG1 and Lnc-DC, which influence its expression or function during inflammation, ER stress, and immune cell differentiation. Moreover, lncRNAs such as LINC00346 and TINCR connect ATF4 signaling to tumor immune evasion, revealing their dual roles in both immune homeostasis and cancer immunology. These findings highlight the ATF4–lncRNA axis as a crucial regulator of immune pathophysiology and a potential target for immunotherapy.

## ATF4–lncRNA interactions in skeletal system diseases

The skeletal system, including bone, cartilage, and associated connective tissues, is frequently affected by mechanical loading, oxidative stress, aging, and inflammatory signals. These stimuli can activate the ISR, where ATF4 plays a vital role in maintaining bone homeostasis, osteoblast differentiation, and response to injury. LncRNAs have emerged as key modulators of ATF4 in skeletal development and pathology.

ATF4 serves as a major transcription factor regulating osteoarthritis (OA) and knee osteoarthritis (KOA) under ER stress conditions. The experiment has proven that by using traditional Chinese medicine Rongjin Niantong formula (RJNTF), ATF4 downregulates lncRNA MGC-Mirg expression in disease models. Under ER stress conditions, the PERK pathway is activated, and ATF4 is upregulated. At the same time, the expression of lncRNA MGC-Mirg is significantly increased, consistent with ATF4. After intervention with taurine deoxycholic acid solution, ER stress was inhibited, and the expression of ATF4 and lncRNA MGC-Mirg was significantly reduced, further supporting the regulation of lncRNA MGC-Mirg by ATF4 (35, 36).

Additionally, through the Wnt-β-catenin pathway, in response to ATF4 signaling, lncRNA H19 and lnc-OAD (Osteogenesis Associated lncRNA during Differentiation) are upregulated. H19 promotes osteogenic differentiation (37), while lnc-OAD modulates adipogenesis via influencing mitotic clonal expansion (38). By contrast, DANCR suppresses osteogenesis by directly repressing ATF4 expression, thereby negatively regulating osteogenic commitment (39).

Conversely, lncRNAs also modulate ATF4 expression or function. HOTAIR, a well-studied lncRNA, regulates ATF4 protein levels through competitive binding to miR-214 underscores the crucial role of HOTAIR in osteoblast function and bone formation. The cellular localization of HOTAIR is pivotal to its function, and HuR plays a key role in regulating the cellular localization of HOTAIR (40).

In steroid-induced avascular necrosis of the femoral head (SANFH), lncRNA MALAT1 upregulates the expression of ATF4 by sponging miR-214, thereby promoting osteogenic differentiation. Specifically, MALAT1 is downregulated in SANFH tissues, while miR-214 expression is upregulated. MALAT1 can directly sponge miR-214, preventing its degradation of ATF4 mRNA, thus increasing the protein level of ATF4 and promoting osteoblast differentiation and function. This mechanism reveals the protective role of MALAT1 in SANFH (41). Another lncRNA, KCNQ1OT1, regulates osteogenic differentiation by targeting the miR-205-5p/RICTOR axis. Silencing KCNQ1OT1 impairs ATF4 expression and reduces bone matrix deposition *in vitro* (42).

In osteosarcoma, LINC00963 enhances tumor cell proliferation by sponging miR-320a, thereby activating ATF4 signaling (43). Another lncRNA SNHG16, its relationship between ATF4 may be mediated by miRNAs. hsa-miR-15b-5p, hsa-miR-93-5p, and miR-20b-5p serve as intermediary molecules, regulating the expression of ATF4 and SNHG16. Specifically, these miRNAs regulate ATF4 expression by binding to its 3’UTR, while SNHG16 may indirectly affect ATF4 expression by sponging these miRNAs (44).

The ATF4–lncRNA axis plays a pivotal role in skeletal system homeostasis, influencing processes such as osteogenesis, adipogenesis, and cartilage integrity under stress conditions. ATF4 can regulate lncRNAs like MGC-Mirg, H19, and lnc-OAD to promote or inhibit bone formation depending on context, while conversely, lncRNAs such as HOTAIR, MALAT1, KCNQ1OT1, LINC00963, and SNHG16 modulate ATF4 expression or activity through ceRNA networks. These bidirectional interactions are closely tied to skeletal pathologies including osteoarthritis, steroid-induced osteonecrosis, and osteosarcoma, offering potential therapeutic targets for degenerative and neoplastic bone diseases.

## Summary of regulatory directions

The regulatory interplay between ATF4 and lncRNAs is complex and context dependent. In some disease settings, ATF4 functions as a transcription factor that induces or represses the expression of specific lncRNAs, often in response to cellular stressors such as ER stress, hypoxia, or nutrient deprivation. In other cases, lncRNAs act upstream of ATF4 by influencing its transcription, translation, or stability through various mechanisms, including the ceRNA pathway and microRNA sponging.

To enhance clarity and avoid conflation of regulatory directions, we have categorized the current literature into two mechanistic groups:

**Table 1A** presents cases in which ATF4 regulates the expression or activity of lncRNAs, with downstream consequences for disease progression.

**Table 1B** highlights lncRNAs that modulate ATF4, either directly or indirectly, thereby shaping ATF4-mediated stress responses or pathological signaling pathways.

This structured summary facilitates a clearer understanding of the bidirectional nature of ATF4–lncRNA regulation and provides a foundation for identifying novel diagnostic markers or therapeutic targets across multiple disease systems.

## *In vitro* and *in vivo* studies elucidating ATF4 mechanisms in systemic diseases

A growing body of research utilizing both *in vitro* and *in vivo* models has significantly advanced our understanding of how ATF4 functions in various disease contexts. These studies provide critical mechanistic insights and validate the functional relevance of the ATF4–lncRNA regulatory network.

In digestive system diseases, HCC cell lines under glucose-deprivation conditions have demonstrated ATF4-dependent upregulation of lncRNAs such as GIMA and LINC01564, promoting autophagy and serine biosynthesis, respectively. These effects were confirmed using reporter assays and knockdown experiments, as well as xenograft mouse models where modulation of ATF4 or its target lncRNAs altered tumor growth and redox balance (11, 16).

In respiratory system research, bleomycin-induced mouse models of pulmonary fibrosis were used to demonstrate that ATF4-induced lncRNA lnc949 alleviates fibrosis by modulating TGF-β/Smad2/3 signaling (23). Additionally, LUAD cell lines and orthotopic tumor xenografts have been employed to study ATF4 activation of LINC00958, validating its contribution to immune escape and tumor progression (22).

In immune system studies, macrophage cultures stimulated with LPS showed increased ATF4 and MIR155HG expression via the PERK–eIF2α pathway. Functional experiments including luciferase reporters and cytokine profiling confirmed this regulation. *In vivo*, mouse models of endotoxemia demonstrated that targeting this axis reduced proinflammatory cytokine levels (30). Similarly, ATF4 modulation by TUG1 and Lnc-DC was validated using diabetic and inflammatory disease models (32, 33).

In skeletal system research, both primary osteoblast cultures and mouse models of osteoarthritis or steroid-induced osteonecrosis have been utilized to examine ATF4’s role in bone remodeling. Studies showed that ATF4-mediated upregulation of H19 and lnc-OAD enhances osteogenesis, while downregulation of MGC-Mirg by Chinese medicine (RJNTF) suppressed endoplasmic reticulum stress and improved cartilage integrity (36, 38). *In vivo* gene knockdown and bone histomorphometry further substantiated the regulatory roles of MALAT1, KCNQ1OT1, and LINC00963 on ATF4 signaling during osteogenesis and bone repair (41–43).

Collectively, these *in vitro* and *in vivo* models offer strong validation for the ATF4–lncRNA axis as a functional and potentially druggable pathway in systemic diseases. Future studies employing CRISPR-based editing and single-cell transcriptomics are expected to deepen our understanding of spatial and temporal dynamics of this regulatory network.

## Therapeutic implications of the ATF4–lncRNA axis

The ATF4–lncRNA regulatory network represents a promising therapeutic target in systemic diseases, particularly those involving chronic stress responses such as cancer, fibrosis, and inflammation. While ATF4 itself is a central transcription factor within the ISR, directly targeting ATF4 may carry a high risk of off-target effects due to its broad expression and essential roles in multiple organs and physiological systems. In contrast, lncRNAs typically exhibit more tissue-specific and disease-contextual expression patterns, making them attractive candidates for precision therapy.

Importantly, therapeutic strategies must consider the directionality of the lncRNA–ATF4 interaction. Upstream lncRNAs (e.g., MEG3, HULC, Gm10768) regulate ATF4 expression or translation, often through ceRNA mechanisms involving microRNA sponging. Targeting these molecules may allow for indirect modulation of ATF4 activity, offering finer control in diseases where ATF4 is aberrantly activated. Conversely, downstream lncRNAs (e.g., LINC01564, ZFAS1, HITTERS) are transcriptional targets of ATF4 and participate in the execution of ATF4-mediated stress adaptation programs such as autophagy, redox regulation, and drug resistance. These downstream effectors may serve as biomarkers for disease progression or as points of intervention to disrupt maladaptive cellular responses.

Furthermore, advances in RNA-based therapies, including antisense oligonucleotides (ASOs), siRNA delivery systems, and CRISPR-based RNA editing, have paved the way for targeting lncRNAs *in vivo* with increasing specificity and safety. Combined with knowledge of lncRNA–ATF4 interactions, these technologies offer potential for tailored therapies that modulate cellular stress responses in a controlled and system-specific manner.

Future studies should explore which lncRNAs are most amenable to therapeutic targeting, their context-dependent roles across tissues, and how their manipulation might synergize with existing treatments such as chemotherapy, immune checkpoint inhibitors, or metabolic modulators.

## Conclusion

The bidirectional regulatory relationship between ATF4 and lncRNAs represents a crucial layer of gene expression control in the context of stress adaptation and disease progression. This review highlights how ATF4, as a central transcription factor in the ISR, transcriptionally activates a subset of lncRNAs that modulate downstream biological functions including autophagy, apoptosis, redox homeostasis, metabolism, and immune signaling. In parallel, a diverse array of lncRNAs modulate ATF4 expression or activity, primarily via microRNA-mediated ceRNA networks, post-transcriptional regulation, or chromatin remodeling.

These lncRNA–ATF4 interactions exhibit system-specific characteristics across digestive, respiratory, immune, and skeletal diseases, underscoring their relevance in conditions such as cancer, fibrosis, metabolic disorders, and immune dysfunction. Notably, the directionality of these interactions carries important implications for therapeutic intervention: upstream lncRNAs may serve as molecular levers to fine-tune ATF4 activity, while downstream lncRNAs may function as disease effectors or biomarkers of ATF4 signaling output.

Given the cell-type and disease-specific expression patterns of lncRNAs, targeting them may offer higher therapeutic precision compared to broadly modulating ATF4 itself. This makes the lncRNA–ATF4 network an appealing framework for developing RNA-based therapies, including antisense oligonucleotides, siRNA approaches, or lncRNA mimics/inhibitors.

Moving forward, future research should focus on functional validation of candidate lncRNAs in *in vivo* models, dissect the spatial-temporal dynamics of ATF4–lncRNA circuits, and evaluate their tractability in therapeutic applications. Integrating transcriptomic, epigenomic, and systems biology data will be essential to unlocking the translational potential of this regulatory network in precision medicine.
